# Deformation of Alkali-Activated Materials at an Early Age Under Different Curing Conditions

**DOI:** 10.3389/fchem.2021.694454

**Published:** 2021-06-08

**Authors:** Mark Češnovar, Katja Traven, Vilma Ducman

**Affiliations:** ^1^ Slovenian National Building and Civil Engineering Institute (ZAG), Ljubljana, Slovenia; ^2^ International Postgraduate School Jozef Stefan, Ljubljana, Slovenia

**Keywords:** alkali-activated materials, slag, drying, autogenous shrinkage, partial surface pressure, curing deformation

## Abstract

The production of alkali-activated materials (AAMs) is known for its environmentally friendly processing method, where several amorphous-rich aluminosilicate material sources combine with an alkali media solution to form solid, ceramic-like materials. In terms of the Si:Al, Na(K):Al, and Na(K):H_2_O ratios, the theory of AAM formation is quite well developed, but some open questions in the technology process remain, especially with regards to the means of curing, where the generation of defects can be persistent. Knowing that deformation is extremely high in the early ages, this study investigates the effects of temperature and moisture on shrinkage behavior within the first 72 h of AA pastes made from ladle (LS) and electric arc furnace (EAF) slag and activated by sodium silicate (Na_2_SiO_3_). The method to determine the deformation of alkali-activated slag-based materials, in terms of both autogenous and drying shrinkage, was based on the modified ASTM C1698-19 standard for the measurement of autogenous shrinkage in cement pastes. Autogenous deformation and strain were measured in four samples, using the standard procedure at room temperature, 40 and 60°C. Furthermore, using an adjusted method, nine samples were characterized for strain and partial surface pressure, while drying at room temperature, 40, or 60°C at a relative humidity of 30 or 90%. The results show that the highest rate of autogenous shrinkage occurred at a temperature of 60°C, followed by drying shrinkage at 60°C and 30% relative humidity, owing to the fact that the rate of evaporation was highest at this moisture content. The study aimed to provide guidance regarding selection of the optimal curing set in order to minimize deformations in slag-based alkali-activated materials. In the present case, curing at a temperature of around 40°C under lower moisture conditions for the first 24 h provided optimal mechanical properties for the slags investigated. The methodology might also be of use for other aluminosilicate sources such as metakaolin, fly ash, and mineral wool–based alkali-activated materials.

## Introduction

The consumption of 33 billion tons of concrete per year (ISO/TC 071 Strategic business plan, Date: 4/14/2016, Version: Final) necessitates the adoption of a new approach toward the production of more environmentally friendly materials. Lately, alkali-activated materials (AAMs) have been studied as a technically acceptable alternative to concrete, with a lower environmental impact due to the incorporation of industrial by-products instead of cement (van Deventer et al., 2012). AAMs possess good properties, similar to concrete or ceramic materials, and can be processed at low temperatures, given that all the thermal pretreatment of precursors is performed when either producing steel (metallurgical slags) or burning coal fuel (ashes) in the steam power plants at the point when the electrical energy is produced (Fernandez-Jimenez et al., 2015). Another advantage of using AAMs is the chance to recover material from landfills, which are often overloaded with industrial by-products, eventually leading to air and water pollution (Natali Murri et al., 2013). Although AAMs could be useful as concrete binders, bricks, or lightweight insulation materials, the physical properties are strongly affected by the source material, alkaline activators, and the processing parameters. Most of the factors that influence the microstructural evaluation and/or mechanical properties of AAMs have, however, already been studied, including the chemical and physical properties of raw materials (Fernandez-Jimenez et al., 2006; Traven et al., 2019; Rajamma et al., 2012), Al/Si (composition) ratios (van Jaarsveld et al., 2002), the activator (Chen et al., 2017), curing regime and aging (Češnovar et al., 2019a; Tran et al., 2019), and the microstructure and phase composition (Khale and Chaudhary, 2007; Ismail et al., 2014). Despite numerous studies regarding the alkali activation process, there is still a lack of knowledge regarding defects in the matrix and methods to evaluate possible causes. Deformation has been more often studied in cement systems (OPC, CAC, CSA, etc.), where autogenous, chemical, and drying shrinkage have all been widely examined. For OPC concrete, it is well known that five types of shrinkage occur: plastic, drying, autogenous, chemical, and carbonation. Plastic shrinkage is an immediate aftereffect of casting, when the water evaporates. Drying shrinkage is the result of dehydration from the gel pores, and autogenous shrinkage is caused by self-desiccation and is a result of higher capillary forces when smaller pores are formed. These influential factors are also relevant for AAMs (Adams and Ideker, 2014). The autogenous shrinkage of cement paste or cured mortar is measured under sealed conditions according to ASTM standard C1698 [ASTM, International]. In the case of cement testing, chemical shrinkage results from hydration of the cementitious materials, and results show the absolute change in volume. It is measured by the standard test method outlined in ASTM C1608 (ASTM, International). Carbonation deformation is the result of CO_2_ penetrating into the concrete from the air. Some of these types of shrinkage have not yet been confirmed in the AAM system. Most of the studies regarding the deformation of AAMs have been made in terms of drying shrinkage, where some authors have found that overall shrinkage is far greater than in the case of OPC. More precisely, in the case study by Matalkah et al. (2019), it was concluded that microstrain was twice as high when investigating a fly ash–based AAMs (using ASTM standard 596). Furthermore, the microstrain in alkali-activated slag-based mortars was reported to be as much as four times higher than in Portland cement (using Spanish standard UNE 80–112–89), when each were tested at room temperature and 50% R.H. (Puertas, et al., 2006). An important factor, which causes substantial deformation, is the evaporation of free water during the preparation of AA pastes and afterward. A small amount of water around the N(K)-A-S-H gel is interstitial, so most of the water from the AA process is not bonded into the AAM matrix, as it is in OPC. Due to the evaporation of water from the hardened AA paste, the instability of the inner structure could cause the formation of pores and cracking (Zuhua, et al., 2009). The higher shrinkage seen in AAMs compared to OPC could also be attributed to the more saturated nature of the material, as was predicted by Ye, who researched the drying of AA slag cement (Ye, et al., 2014). Current studies show that autogenous shrinkage is an important factor in the AAM process because it develops quickly at the point when strain on the material is low, which could be the cause of microcracking (Nedeljković et al., 2018; Jensen and Hansen, 2001). In a study where samples were cured under controlled humidity conditions, the autogenous expansion of AAMs at certain curing stages was detected, so such a trend cannot be the result of autogenous deformation according to the desiccation theory for OPC, where the expansion results from the formation of ettringite and the subsequent reincorporation of water back into the matrix (Mobili et al., 2016). Li also extensively studied the correlation between deformation and reaction processes in a metakaolin geopolymer system, where three stages of chemical shrinkage occur (Li, et al., 2019). Chemical shrinkage or expansion is where the absolute change in volume of a material occurs as a result of chemical reactions (Lura et al., 2003). Within the first few hours, the dissolution of aluminosilicates causes shrinkage; after approximately 8 h, the formation of Al-rich products consisting of gels takes place, along with expansion, which is followed by shrinkage when the Al species are further polymerized with available silicate oligomers to produce a Si-rich network (Li, et al., 2019). Li recently proposed a new hypothesis regarding the autogenous shrinkage of AAMs, where authors suggest that besides the “self-desiccation,” the reduction of steric–hydration force due to the significant consumption of ions also induces a certain amount of shrinkage (Li et al., 2020). Curing methodologies have a strong impact on the deformation of AA pastes, which affects the final strength properties of AAMs (Mastali et al., 2018; Gudmundsson, 2013). Moreover, the flexural and compressive strengths can be increased by simply adopting an appropriate curing regime (Češnovar et al., 2019a). The temperature is especially important when the AA paste is setting, as this is the point at which chemical reactions such as dissolution, gelation, and polymerization take place. Some studies have been performed to try to prevent excessive shrinkage or microcracking in AAM systems. One study (Kuenzel et al., 2012) reported the effect of the alkali activator on the shrinkage of metakaolin, where an increased Na^+^ content increased the need for structural water in order to prevent shrinkage, whereas replacing Na^+^ with K^+^ decreased the amount of water required. Collins and Sanjayan (2001) studied drying shrinkage in AAMs and concluded that compressive strength decreases when the evaporation of water causes microcracking and the formation of a capillary network. Another way to prevent high rates of water evaporation is to cure the AA samples in a moist environment with an R.H. greater than 99% (Vlček, et al., 2014). The overall deformation in AAMs is mostly a result of chemical reactions and the evaporation of water from the AA pastes (i.e., drying). At room temperature and low humidity, as incorporated water evaporates from the paste during the AA process, the volume of AA bodies reduces and cracking occurs owing to capillary pressure between the wet and dry micropore areas (Mastali et al., 2018; Li et al., 2018).

The goal of the present study was to assess deformation during the alkali activation of ladle and electric arc furnace slag and to further investigate the effect of the curing regime, including temperature, moisture, and time, on shrinkage in order to identify possible causes of deformation.

## Materials and Methods

For this experimental study, we used electric arc furnace and ladle slag as precursors for the alkali activation process, with sodium silicate as the activator. Both slags were sourced from the Slovenian metallurgical industry. The chemical compositions of both slags analyzed by an X-ray fluorescence instrument (XRF-Thermo Scientific ARL Perform X, United States) are shown in Table 1.

**TABLE 1:** Chemical compositions of Slag A and Slag R used for the preparation of AAMs (in wt%).

	Na_2_O	MgO	Al_2_O_3_	SiO_2_	K_2_O	CaO	Cr_2_O_3_	MnO	Fe_2_O_3_	LOI
Slag A	0.13	14.87	8.54	21.05	0.17	20.87	3.76	2.24	11.37	14.15
Slag R	0.28	23.25	5.20	13.69	0.14	27.85	0.18	0.62	4.64	20.47

The quantitative determination (Rietveld analysis) of mineral phases *via* X-ray diffraction analysis (XRD, Malvern PANalytical Empyrean, NL and United Kingdom) confirmed that both slags contained a high percentage of amorphous phase (55 wt% for slag A and 35 wt% for slag R).

**FIGURE 1:**
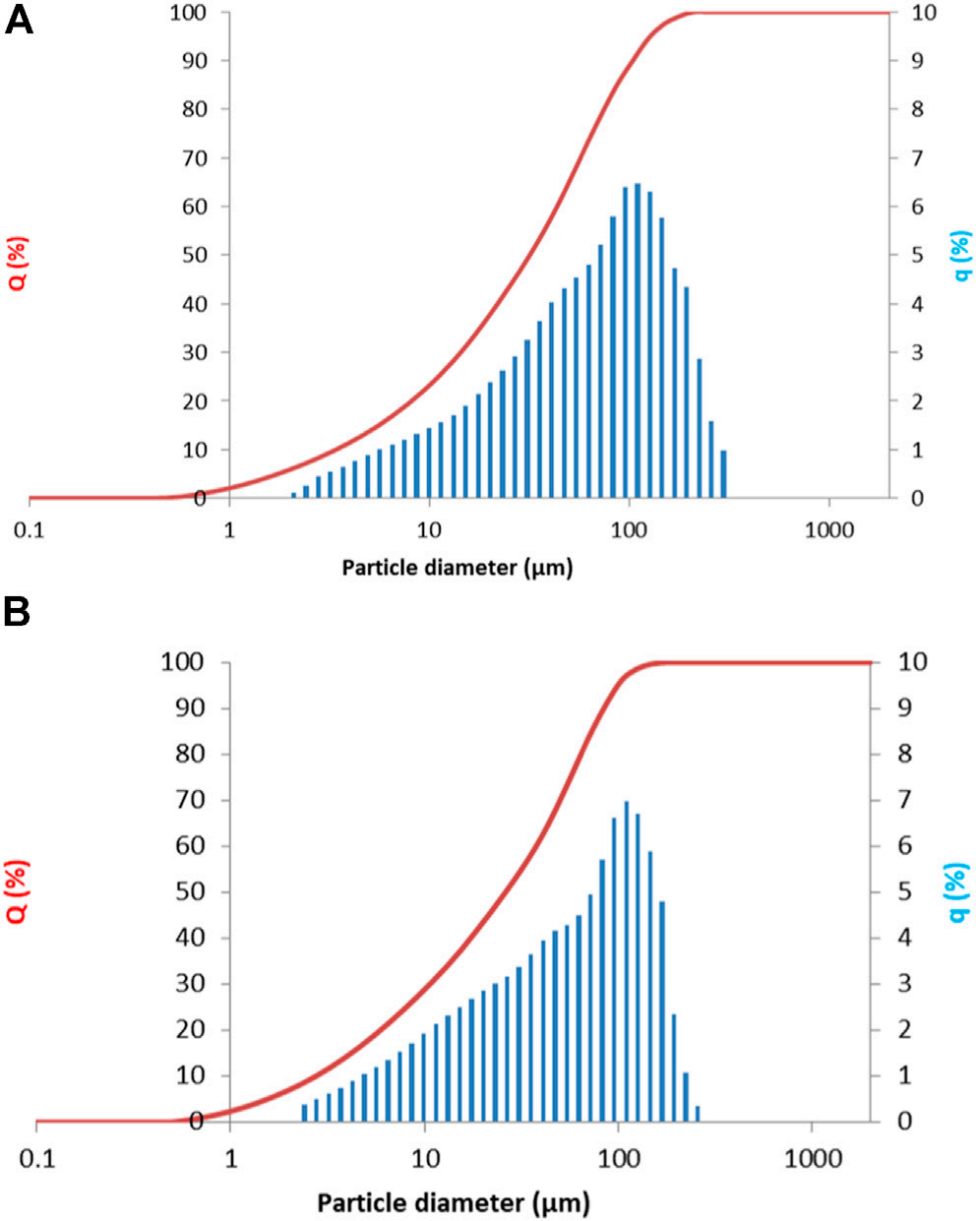
Particle size distribution plotted as a cumulative curve (red line) and the particle size distribution curve (histogram in blue) for **(A)** Slag A and **(B)** Slag R.

Prior to alkali activation, slags were milled and sieved to below 125 µm. Particle size distribution was measured using a Microtrack Sync (United States, Pennsylvania) laser granulometer in isopropyl alcohol with ultrasound. The particle size distribution was d_10_ = 2.02, d_50_ = 22.90, and d_90_ = 88.00 µm for Slag A, and d_10_ = 2.61, d_50_ = 23.42, and d_90_ = 81.29 µm for Slag R. The cumulative curve is presented in Figure 1.

BET-specific surface area by nitrogen adsorption at 77° K has been determined for both slags by means of Micromeritics ASAP 2020 (Micromeritics, Norcross, GA, United States), and it amounted 6.79 and 7.06 m^2^/g for slag A and slag R, respectively.

**FIGURE 2:**
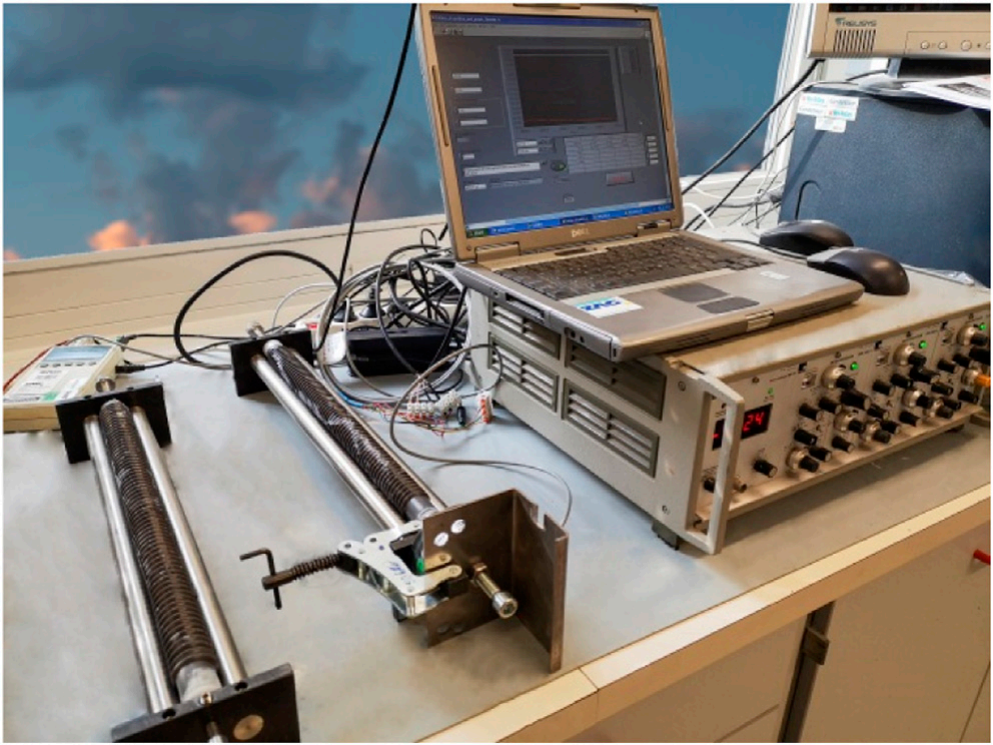
Automated measuring system for autogenous shrinkage and force.

**FIGURE 3:**
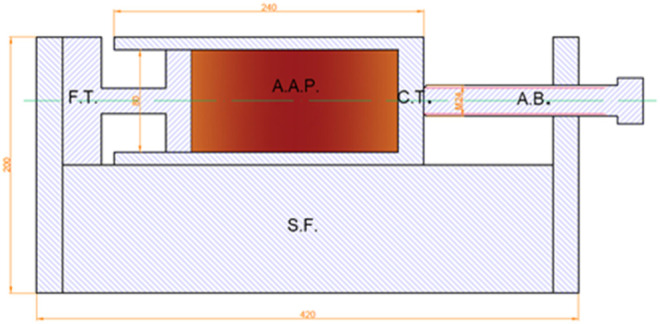
Arrangement for measurement of the partial drying pressure. F.T. is a force transducer, A.A.P. is the alkali-activated paste (sample), S.F. is a steel frame, C.T. is a cardboard tube, and A.B. is an adjustment bolt.

The development of AAM mixtures based on these precursors was already discussed elsewhere (Češnovar et al., 2019b), whereas a slightly modified optimal mixture was used in the present study containing slag A and slag R mixed in a 1/1 ratio and activated with 33 wt% Na_2_SiO_3_ (commercial Sodium silicate Crystal 0112 in a 2:1 SiO_2_/Na_2_O ratio; Tennants Distribution Limited, United Kingdom) and 1 wt% NaOH (Donau Chemie, Ätznatron Schuppen, EINECS 215–785–5, Wien, AT); 654 g of precursors was activated with 327 g of Na_2_SiO_3_ (33 wt%) and 10 g of solid NaOH (1 wt%). To aqueous Na_2_SiO3, pregrinded solid NaOH was added and mixed until it was completely dissolved. Afterward, the precursor was gradually added to the activators mixture, and pastes were homogenized in a laboratory mixer for 5 min and then cast and sealed in a corrugated tube (420 mm × ø 29 mm), as described in the standard method for unrestrained measurement outlined in ASTM C1698 to measure autogenous shrinkage. The same mixtures were cast in microfiber clothing tubes (300 mm × ø 30 mm) in order to measure the drying shrinkage under saturated conditions. The sealed mixtures were cured under different conditions, one at room temperature (Figure 2), as suggested by the ASTM standard C1698 and the others at temperatures of 40 and 60°C, by heating the sealed specimens in a laboratory heat chamber, in order to determine the effect of heat energy on shrinkage development. Further mixtures were exposed to saturated conditions at 90% R.H. and 40% R.H. at room temperature (Figure 3), 40 or 60°C. Measurements of autogenous and drying shrinkage were taken automatically by a linear variable differential transformer (LVDT) for 72 h. Force was measured using a 30 N force transducer with an internal measuring amplifier (A.S.T. Germany). The initial force of 15 N was continued to provide measurements of reaction in the positive or negative direction. This force was then deducted from the value measured. After 72 h, the samples were prepared to measure flexural and compressive strength.

The strain was calculated according to Eq. 1.

$$\varepsilon =\frac{\Delta l}{l}\left(\%\right),$$ (1)

where Δ*l* is the change in length of material under stress and *L* is the original length of the specimen measured immediately after molding.

Surface partial pressure was calculated according to Eq. 2.

$$\delta =\frac{\text{F}}{\text{A}}\left(Pa\right),$$ (2)

where F is the force acting on the surface of the material, measured on the longer edge due to the deformations and A is the area where the force was measured.

Microstructural analysis of the polished surfaces of hardened AAMs was performed using a JEOL scanning electron microscope (SEM) in back-scattered electron image mode (JSM-IT500 LV, Jeol, Tokyo, Japan) in a low vacuum. Quantitative analysis was performed using energy-dispersive spectroscopy (EDS: Oxford instruments, Abingdon, United Kingdom), using the Aztec software platform.

## Results and Discussion

### Deformation of the AAM Pastes

**FIGURE 4:**
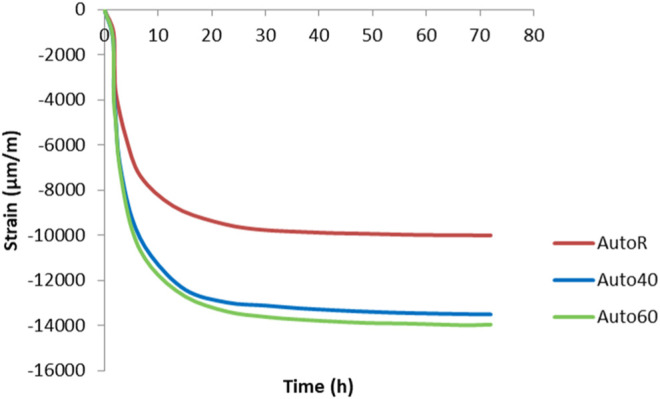
Autogenous shrinkage measured by a standard procedure at room (AutoR) and elevated temperatures (Auto40 is at 40°C and Auto60 is at 60°C).

Deformation of the AAM pastes through autogenous shrinkage for the first 72 h of curing is presented in Figure 4. The measuring procedure in standard C1698-09 was followed, which specifies curing at room temperature for 72 h, with parallel measurements made at elevated temperatures of 40 and 60°C in order to evaluate the deformation caused at higher temperatures. Shrinkage starts immediately, as soon as measurement commences, but is slow for the first hour. This could be attributed to the chemical expansion (Li et al., 2019). After this time, the autogenous shrinkage is greater, and deformation continues rapidly within the first 10 h, at which point it slows down to the rate of −8,000 μm/m in the specimen dried at room temperature. Shrinkage is greater in the specimens cured at elevated temperatures (40 and 60°C). As Altan and Erdoğan. (2012) showed, hydration and therefore rearrangement of the microstructure are greatly accelerated at an elevated temperature (Altan and Erdoğan, 2012). In all specimens, the deformation slows down after the first 24 h and does not change much over the subsequent two days of curing.

**FIGURE 5:**
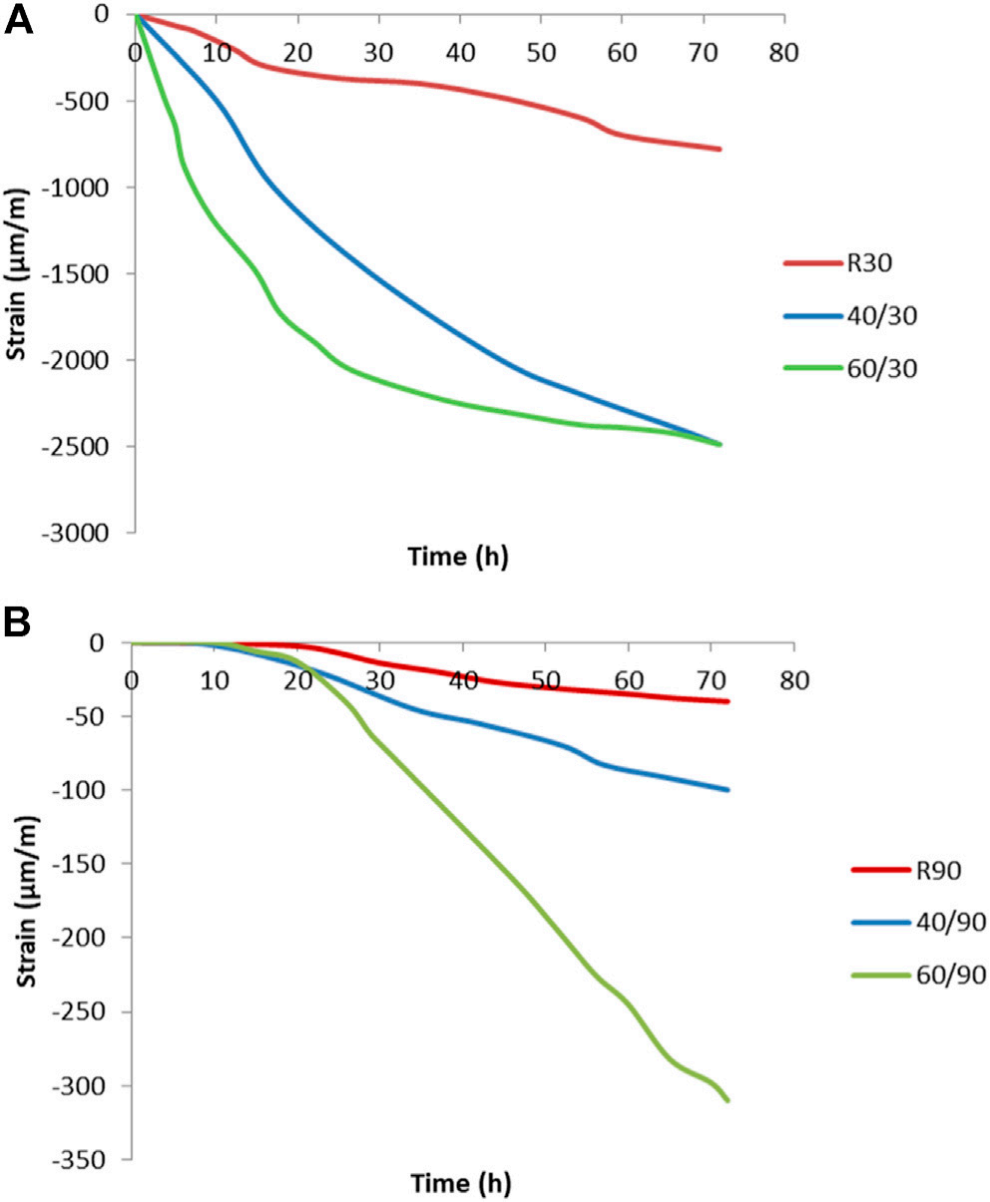
The results of early-age drying shrinkage measured at **(A)** 30% R.H., where R is room temperature, 40 is at 40°C, and 60 is at 60°C and **(B)** at 90% R.H. at room temperature (R), 40°C (40), and 60°C (60).

The shrinkage caused by early age drying shrinkage, where the volume is changed due to the evaporation or consumption of moisture, is presented in Figures 5A,B.

At all three temperatures, the shrinkage observed under conditions of 30% R.H. (relative humidity) was approximately 10 times higher than that in saturated conditions of 90% R.H. This is due to increased water evaporation in the specimens at the lower relative humidity. The strain development at 90% R.H. is much slower, due to the slower evaporation of moisture, or even the consumption of water from the external environment, which leads to a low change in volume. As can be seen in Figure 5, the influence factor of moisture is 6.5 times higher than the influence of an elevated temperature.

**FIGURE 6:**
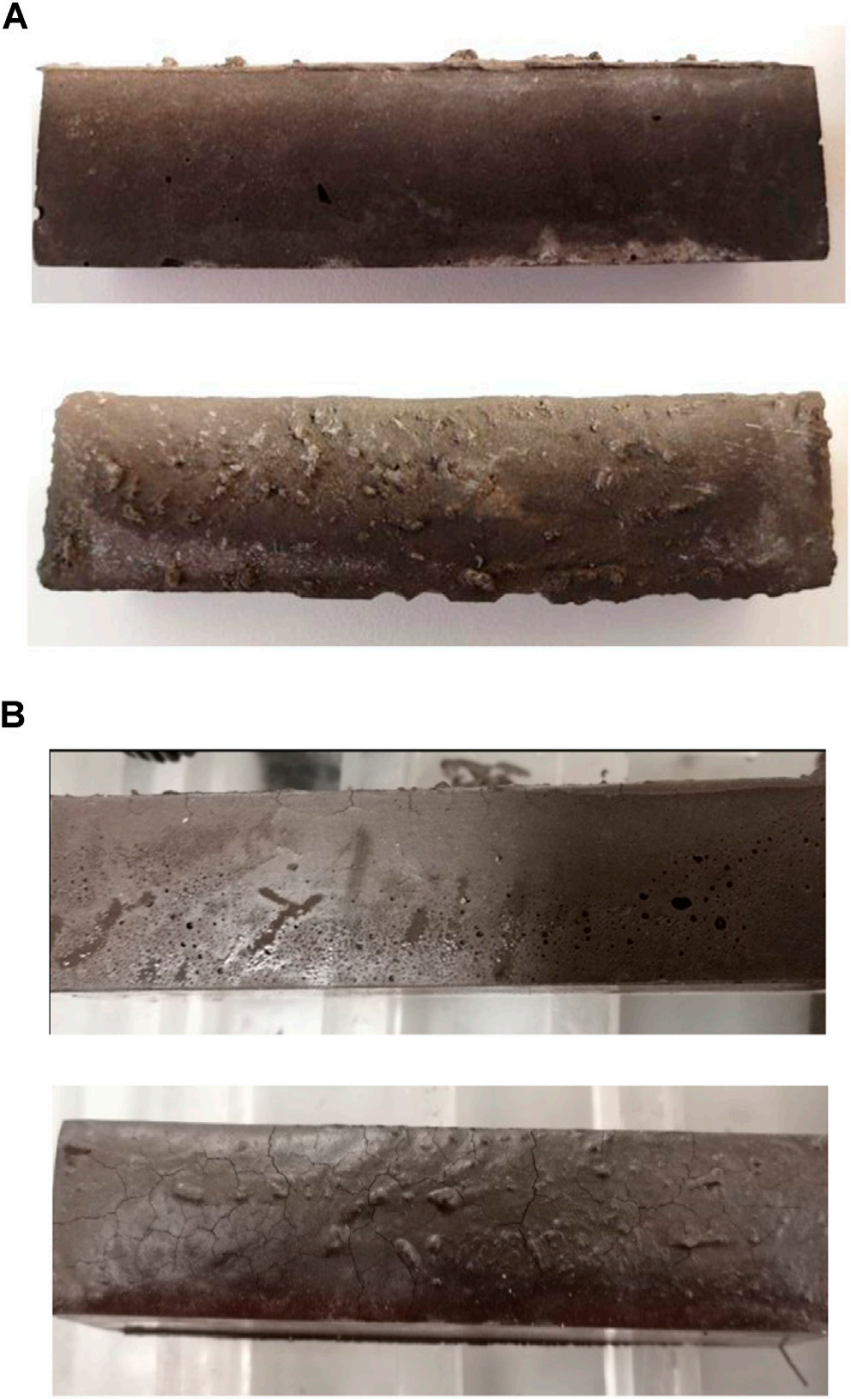
80 cm^3^ × 20 cm^3^ × 20 cm^3^ samples at room temperature and **(A)** 30% R.H. or **(B)** 90% R.H.

Curing under saturated conditions also causes cracks on the samples. Cracks are formed on the surface when the specimen is cured at 90% R.H., as shown in Figure 6B, whereas there is no visible crack formation when curing at 30% R.H. (Figure 6A).

### Surface Partial Pressure

**FIGURE 7:**
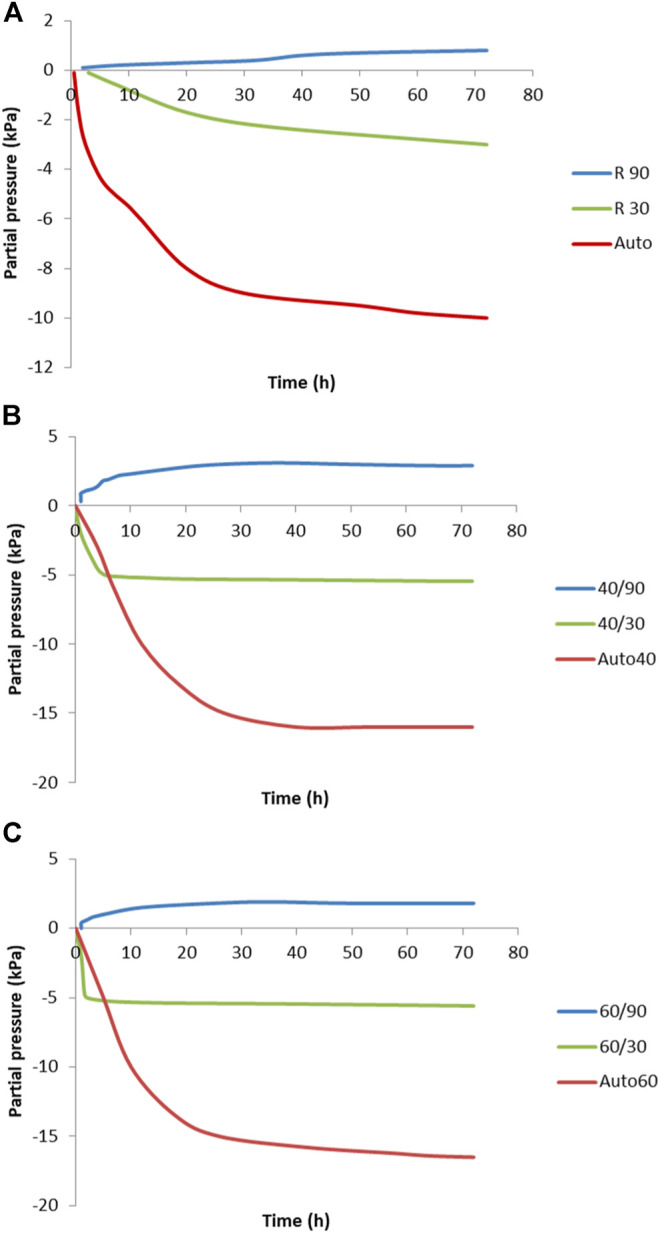
Partial pressure as a function of curing temperature at **(A)** room temperature, **(B)** 40°C, and **(C)** 60°C at both 30 and 90% relative humidity.

Figure 7 shows the partial pressure measured on the surface of the specimens cured under different conditions. As seen, the lowest pressure is measured on the samples under sealed conditions, where autogenous shrinkage is the highest. Studies from Li suggest that when the concentration of ions is stable, the reaction is faster. The autogenous shrinkage is greater due to increased pore pressure over the same period, as has also been confirmed by other studies by Cartwright and Li (Cartwright et al., 2014). High pore pressure is generated in the AAMs, which increases the autogenous shrinkage and induces self-desiccation, especially at an early age (Li et al., 2020).

To determine the factors other than pore pressure that affect shrinkage, specimens were cured in highly saturated conditions (90% of R.H). In this environment, the shrinkage was very low, but the partial pressure was higher than in all other experiments regardless of the environmental temperature as shown in Figure 7. One of the reasons for this could be a lower surface tension, causing lower shrinkage or even expansion. Expansion was not confirmed in this study, due to the short period of time over which deformation was measured. This prediction follows the suggestion that the cause of low shrinkage in cement systems under saturated conditions is the formation of ettringite crystals, which reduces the capillary forces thus increasing pressure on the microstructure where the absence of capillary forces increases the pressure on the microstructure (Li et al., 2020). Thomas studied the dimensional stability of AA concrete during aging and heat curing and concluded that the higher drying shrinkage was due to the greater pore capillarity stress. A higher total porosity increases the pore volume and water loss (Thomas et al., 2017). In the case of AAMs, the higher porosity, together with a higher pore volume, results in greater water loss. When the relative humidity during curing is sufficiently high, water stays in the pore structure and minimizes the capillary stress. Nevertheless, AAM systems differ to those from cement, as due to the large amounts of ions in the initial solution the interstitial space counterbalances the attractive forces between gel particles and resists densification of the gel structure (Ye and Radlińska, 2016). Figures 7A–C show the same trends, despite the curing conditions being different; in particular, the higher temperature does not have any particular effect other than accelerating the progression in the early stages of curing. Ye et al. suggested that in chemical reactions where the concentrations of ions such as SiO_3_^-2^ decrease, these reduced concentrations lead to a reduction in the steric–hydration forces. The end result is that the gel particles around pores become closer, thus causing a rearrangement of the gel structure (Ye and Radlińska, 2016).

### Mechanical Properties

**FIGURE 8:**
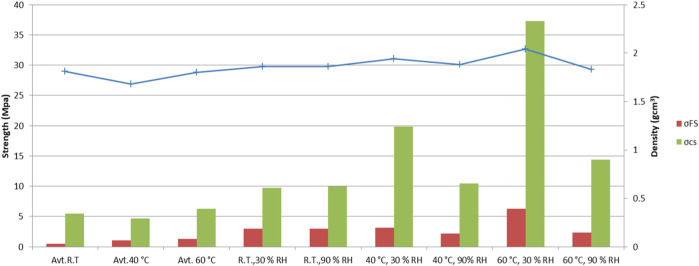
Flexural strength, compressive strength, and density.

Figure 8 presents the flexural strength, compressive strength, and densities of the specimens treated under various curing conditions. As expected, different curing conditions significantly influence the mechanical properties through a variety of deformations and cracks. At room temperature, the effect of different moisture conditions on strength or density is not evident, whereas at a higher temperature, the lower moisture content could be more than double the strength. Despite a strain of approximately 10.000 μm/m, and a partial pressure of 15 kPa, in the case of curing under sealed conditions, the densities (1.55–1.75 g/cm^3^) and strengths did not develop as well as in the specimens treated under low moisture conditions. This could be caused by the larger pore volume, leading to a higher pore pressure and the formation of cracks and unbound water, resulting in low densities. Furthermore, the highest density was around 2 g/cm^3^, with flexural and compressive strengths of 6 and 37 MPa, respectively, when samples were cured at low humidity (30%) and a temperature of 60°C. From the results shown, it was concluded that the partial pressure was negative and near zero at a low relative humidity. The initial densification of AAMs should therefore be slow, preferably at an R.H. of 45% or less in order to employ surface tension and avoid disjoining pressure (Mehta and Monteiro, 2014), and at low temperatures, as was presented in previous studies regarding the influence of various curing temperatures (Češnovar et al., 2019a).

### Microstructure

**FIGURE 9:**
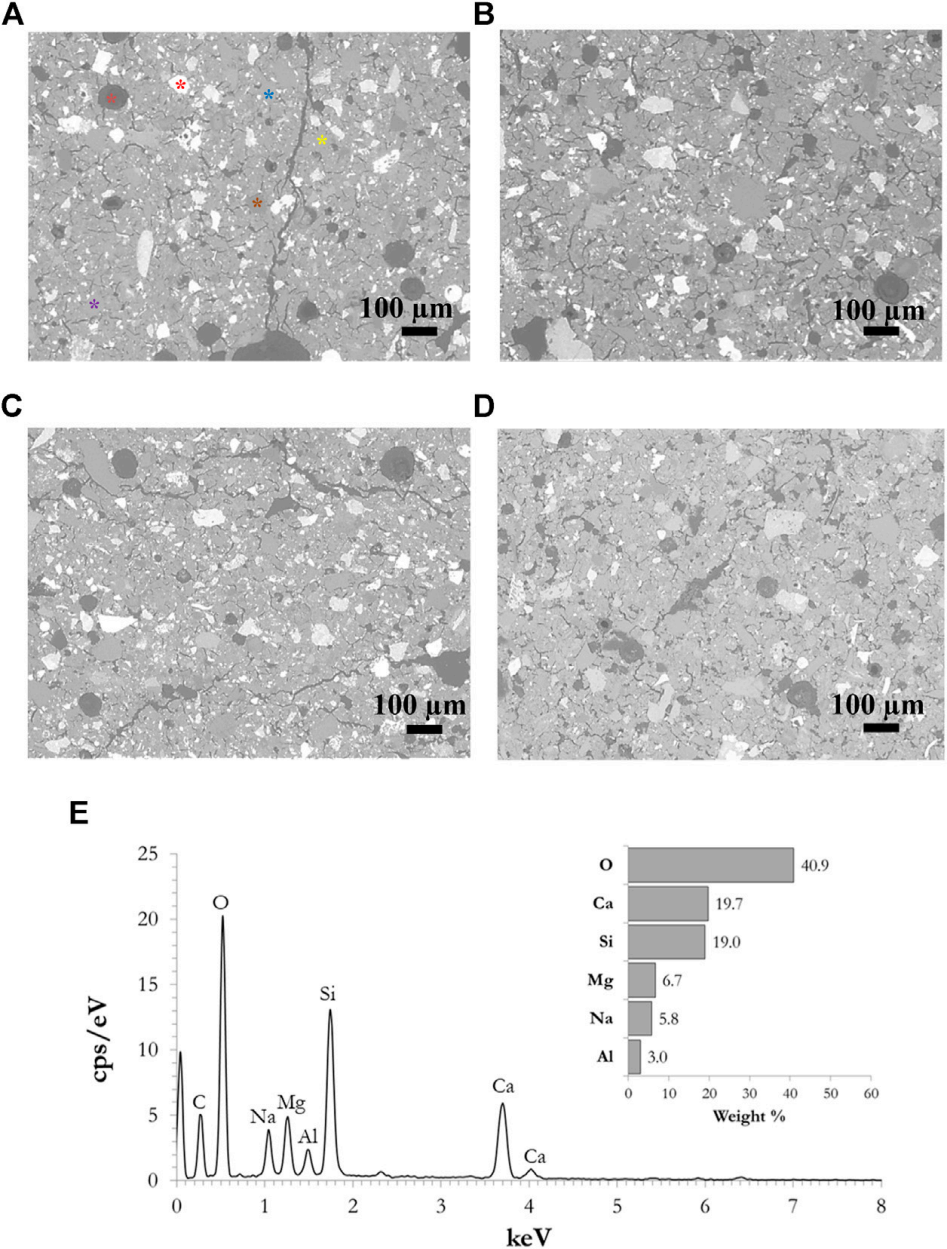
SEM micrographs of samples cured: **(A)** when sealed at room temperature, **(B)** when sealed at 60°C, **(C)** at room temperature and 30% R.H., **(D)** at 60°C and 30% R.H., and **(E)** a representative EDS spectrum of the AAM gel [taken from the position marked by the yellow star in Panel **(A)**].

The microstructure of the samples was investigated following curing under different conditions. SEM micrographs of selected sealed samples cured at room temperature and at 60°C, as well as samples cured at the same two temperatures unsealed at 30% R.H., are presented in Figures 9A–D. More or less, the same pattern can be observed in all four samples (as well as in all the other samples examined which are not presented in Figure 9). Many microcracks are also present in the samples cured in conditions of high humidity, which would not be expected to the same degree in the case where low humidity conditions were applied. In all cases, the presence of spherical pores was also observed (Figure 9A, orange star), with diameters ranging from a few microns up to 200 µm. The pores are dark gray in color since they are filled with epoxy, which was used during the preparation of the cross sections and was also absorbed into the matrix due to the porosity of the material. Thus, the presence of carbon, which belongs to the epoxy resin, is also observed in the EDS spectrum (Figure 9E). This is not taken into account in the calculation of the gel’s composition. Many unreacted particles are observed in the structures, meaning that they did not dissolve in the alkalis during the alkali activation process and thus do not form part of the alkali-activated gel but represent only filling in the matrix. Representative unreacted particles are marked in Figure 9A, where an Fe-rich particle is marked by a red star, Mg-rich by purple, Ca-rich by blue, and an Si-rich particle by a green star. The EDS spectrum of the alkali-activated gel is presented in Figure 9E, taken from the spot marked by a yellow star in Figure 9A. The gel composition was comparable in all the samples investigated, suggesting that deformations during the curing stage do not significantly affect the chemical processes during the alkali activation process.

## Conclusion

Autogenous (ASTM Standard C 1698–09) and early-age drying shrinkage measurements were employed in this study to investigate the effect of curing on the deformation of alkali-activated ladle and electric arc furnace slag. The focus was on the deformation of specimens in different environments, varying in terms of temperature and moisture. Autogenous and drying shrinkage were measured in order to evaluate the deformation of specimens following alkali activation. At room temperature, the shrinkage (autogenous deformation) was highest in the samples exposed to a lower relative humidity. The same trends are shown at higher temperatures of 40 and 60°C, where rapid shrinkage is seen in specimens from the point at which curing commences. This could be attributed to the chemical reaction, where the reaction kinetics is higher.

The early-age drying shrinkage was measured by the same principle in unsealed specimens exposed to different moisture conditions, that is, saturated at 90 and 30% R.H. Very low shrinkage occurred in specimens treated under saturated conditions, as the result of the higher partial pressure measured in this study. Expansion could occur under these conditions, but it was not confirmed due to the short period of measurement.

The mechanical properties of specimens are strongly influenced by the curing conditions. The strength of specimens cured at a lower moisture and higher temperature is double than those cured at room temperature. Specimens cured under sealed conditions exhibited high strain and low partial pressure and did not develop good strength, which could be due to the fact that the large pore pressure caused microcracks and unbound water in the material matrix, leading to low density and strength.

Microstructural evaluation presents pores and cracks in all samples, but they differ in size and distribution. EDS analysis revealed the gel composition to be comparable in all samples investigated, suggesting that deformations during the curing stage do not significantly affect chemical processes during the alkali activation process.

## Data Availability

The original contributions presented in the study are included in the article/Supplementary Material, and further inquiries can be directed to the corresponding authors.
